# Phytochemical Characterization for Quality Control of *Phyllostachys pubescens* Leaves Using High-Performance Liquid Chromatography Coupled with Diode Array Detector and Tandem Mass Detector

**DOI:** 10.3390/plants11010050

**Published:** 2021-12-24

**Authors:** Chang-Seob Seo, Kwang-Hoon Song

**Affiliations:** 1 KM Science Research Division, Korea Institute of Oriental Medicine, Yuseong-daero 1672, Yuseong-gu, Daejeon 34054, Korea; csseo0914@kiom.re.kr; 2 KM Convergence Research Division, Korea Institute of Oriental Medicine, Yuseong-daero 1672, Yuseong-gu, Daejeon 34054, Korea

**Keywords:** quality assessment, bamboo, LC–MS/MS, method validation

## Abstract

*Phyllostachys pubescens* leaves are cultivated in a number of Asian countries and have been used for antipyretic and diuretic effects since ancient times, especially in Korea. The purpose of this study was to develop and validate of analytical method for quality control of *P. pubescens* leaves using high-performance liquid chromatography with diode array detector (HPLC–DAD) and liquid chromatography with tandem mass spectrometry (LC–MS/MS) detection. HPLC–DAD analysis was conducted with a Gemini C_18_ column, and distilled water–acetonitrile (both with 0.1% (*v/v*) formic acid) mobile-phase system. For the LC–MS/MS analysis, all markers were separated with a Waters ACQUITY UPLC BEH C_18_ column and gradient flow system of distilled water containing 0.1% (*v/v*) formic acid and 5 mM ammonium formate–acetonitrile. In both method, major components were detected at 2.13–11.63 mg/g (HPLC–DAD) and 0.12–19.20 mg/g (LC–MS/MS). These methods were validated with respect to linearity (coefficient of determination >0.99), recovery (95.22–118.81%), accuracy (90.52–116.96), and precision (<4.0%), and were successfully applied for the quantitative analysis of *P. pubescens* leaves.

## 1. Introduction

*Phyllostachys pubescens* Mazel (Moso bamboo, family; Gramineae), is widely distributed in Asia, Africa, and Latin America and is one of the bamboo species, e.g., *P. nidularia*, *P. sulphurea*, *P. spectabilis*, *Dendrocalamus giganteus*, *Sara argenteastriatus*, *Pseudosasa japonica*, *Pleioblastus fortunei*, and *Lophatherum gracile* [1,2]. Components such as flavonoids (isoorientin, isovitexin, orientin, and vitexin), coumarins (skimin, scopolin, umbelliferone, psoralen, and xanthotoxin), phenylpropanoids (*p*-coumaric acid and chlorogenic acid), and polysaccharides (rhamnose, arabinose, mannose, glucose, and galactose) have been reported to be present in leaves of bamboo species [1,2,3,4,5,6,7]. Among the various components of bamboo leaves, flavonoids, especially flavone *C*-glucosides such as orientin, isoorientin, isovitexin, and vitexin, are the major compounds [8,9].

Studies on the biological activity of bamboo leaves have identified anticancer, anti-inflammatory, antibacterial, antiviral, diuretic, and antiobesity effects [2,3,10]. Among the many activities of bamboo species leaves, those of *P. pubescens* have been reported to have antifungal, antiobesity, antioxidant, and anticoagulant effects [11,12,13]. Vinpocetine, isolated from leaves of *P. pubescens*, has also been reported to prevent osteoblast apoptosis and osteonecrosis of the femoral head [14]. We recently investigated the effect of extracts from *P. pubescens* leaves on SRD5A2 gene expression in human prostate cell lines and an animal model of testosterone-induced benign prostatic hyperplasia [15].

A range of analysis methods has been reported for phytochemical profiling and quantification of *P. pubescens* leaves; these include methods based on high-performance liquid chromatography (HPLC) with ultraviolet or diode array detection (DAD), thin-layer chromatography, liquid chromatography–mass spectrometry (LC–MS), Fourier transform infrared spectroscopy, and gas chromatography–mass spectrometry (GC–MS) [1,2,3,4,5,6,8,16,17]. However, no simultaneous assay for the analysis of flavonoids and phenylpropanoids in *P. pubescens* leaves using liquid chromatography–tandem mass spectrometry (LC–MS/MS) has been developed and validated.

GC, GC–MS, HPLC, and LC–MS systems have long been used for qualitative and quantitative analysis of traditional Chinese medicine (TCM), traditional Korean medicine (TKM), and Kampo medicine (KM) such as herbs or herbal products. Although HPLC systems are the most commonly used analytical instrument for quantitative analysis of TCMs, LC–MS systems can be used to rapidly and accurately detect large numbers of chemicals, and the latter approach is being used more frequently because of the complexity of TCM and various characteristics of phytochemicals [18,19].

The purpose of the present study was development and validation of two rapid, accurate, and sensitive quantification methods (HPLC–DAD and LC–MS/MS) to determine the six marker components (chlorogenic acid, isoorientin, orientin, isovitexin, vitexin, and *p*-coumaric acid) for quality control of *P. pubescens* leaves.

## 2. Results

### 2.1. HPLC–DAD Analysis

#### 2.1.1. Optimization of HPLC–DAD Analytical Conditions

**Figure 1:**
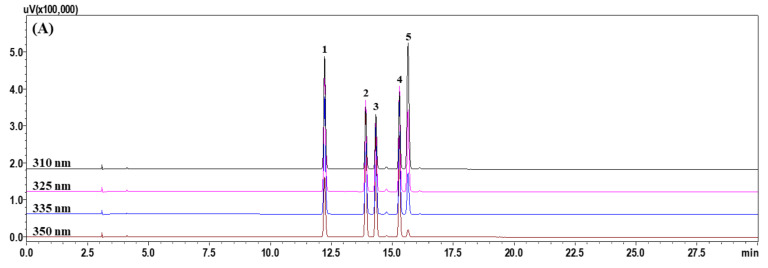

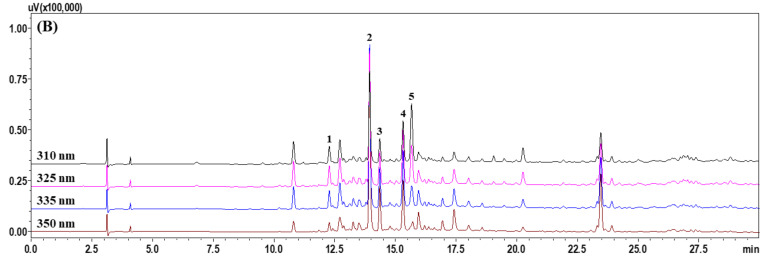
Representative HPLC chromatograms of (**A**) mixture of five marker standard compounds and (**B**) an extract from *P. pubescens* leaves. Peaks eluted in the order chlorogenic acid (**1**), isoorientin (**2**), orientin (**3**), isovitexin (**4**), and *p*-coumaric acid (**5**) at 12.24, 13.92, 14.34, 15.30, and 15.66 min, respectively.

For the HPLC–DAD study, Gemini C_18_ (Phenomenex, Torrance, CA, USA), SunFire C_18_ (Waters, Milford, MA, USA), Xbridge C_18_ (Waters, Milford, MA, USA), Capcell Pak UG80 (Shiseido, Tokyo, Japan), and Quasar SPP C_18_ (PerkinElmer, Seoul, Korea) columns were tested with a range of column temperatures (30, 35, and 40 °C), flow rates (0.8 and 1.0 mL/min), and gradient composition of mobile phase (distilled water–acetonitrile), and acids (0.1% formic acid and 0.1% phosphoric acid). Satisfactory separation of all marker compounds was achieved with a Gemini C_18_ column, 0.1% formic acid, and column temperature of 40 °C, as shown in Table S1; the five markers eluted within 20 min (chlorogenic acid, isoorientin, orientin, isovitexin, and *p*-coumaric acid at 12.24, 13.92, 14.34, 15.30, and 15.66 min, respectively; Figure 1).

#### 2.1.2. Method Validation of the Developed HPLC Assay

The system suitability parameters capacity factor (*k*′), selectivity (*α*), theoretical plate number (*N*), resolution (*Rs*), and tailing factor (*Tf*) were tested to assess the stability of the measurements and operation of the HPLC system; as shown in Table S2, the corresponding values were 2.94–4.06, 1.03–1.19, 399752–722500, 2.62–14.09, and 1.051.10. As shown in Table 1, the coefficient of determination (*r*^2^) values of the five marker components were 1.0000, indicating excellent linearity. By using Equations (1) and (2) (Section 4.6), limit of detection (LOD) and limit of quantification (LOQ) values were calculated to be 0.03–0.10 μg/mL and 0.09–0.29 μg/mL, respectively (Table 1). The recovery (%) of the five marker analytes was 95.22–101.29% from Equation (3) (Table 2), and precision of relative standard deviation (RSD, %) 1.50% was calculated by Equation (4) (Table 3, Tables S3 and S4). These data confirmed that the HPLC–DAD method developed in this study can be used to rapidly and simultaneously analyze the five marker components in *P. pubescens* leaves extract.

#### 2.1.3. Quantification of the Five Markers in *P. pubescens* Leaves Samples by HPLC–DAD Analysis

The optimized HPLC–DAD assay was successfully applied to simultaneous quantitation for quality control of *P. pubescens* leaves. Table 4 shows the content of each marker in samples of freeze-dried *P. pubescens* leaves; the concentrations of the five marker components were determined to be 1.71–11.63 mg/g.

### 2.2. LC–MS/MS Analysis

#### 2.2.1. Optimization of LC–MS/MS Analytical Conditions

**Figure 2:**
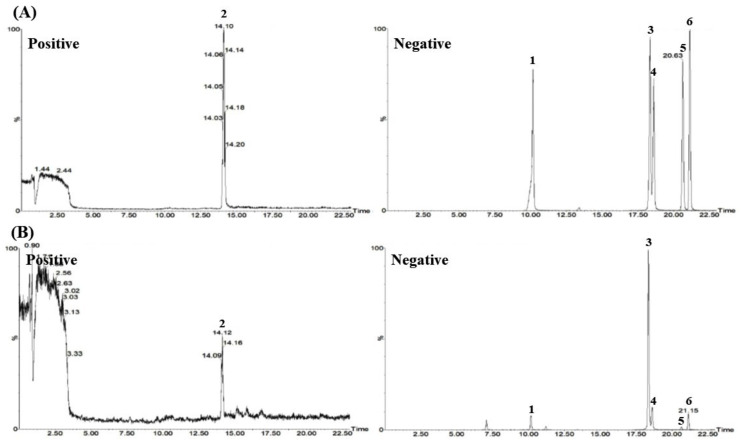
Total ion chromatograms of mixtures of the six marker components (**A**) and 80% ethanol extract of the *P. pubescens* leaves (**B**) were measured by LC–MS/MS MRM in positive and negative ion modes. Chlorogenic acid (**1**), *p*-coumaric acid (**2**), isoorientin (**3**), orientin (**4**), vitexin (**5**), and isovitexin (**6**).

Optimal analysis conditions were screened for quantitative analysis of *P. pubescens* leaves with the LC–MS/MS system (Waters, Milford, MA, USA) combined with a Waters ACQUITY ultra-performance liquid chromatography (UPLC) I-Class system and Xevo TQ-XS tandem quadrupole mass spectrometer. In this system, six markers were separated and quantified with an ACQUITY UPLC BEH C_18_ column (2.1 mm × 100 mm, 1.7 μm, Waters, Milford, MA, USA) maintained at 45 °C under gradient elution conditions of distilled water, containing 0.1% (*v/v*) formic acid and 5 mM ammonium formate, and acetonitrile as a mobile phase. Simultaneous analysis using the multiple reaction monitoring (MRM) mode (Table 5 and Table S5) identified all six marker components within 22 min. Among these markers, *p*-coumaric acid was detected at *m/z* 165.0 in positive ion mode ([M + H]^+^), and the other five marker components, chlorogenic acid, isoorientin, orientin, vitexin, and isovitexin, were detected at *m/z* 353.2, 447.2, 447.2, 431.2, 431.2, respectively, in the negative ion mode ([M − H]^−^) (Figure 2 and Figure S1).

For the simultaneous analysis, *P. pubescens* leaves using the six markers with the established LC–MS/MS method, the MRM transition, the precursor ion (Q1), and product ion (Q3) of each marker were set, as shown in Table 5. The Q3 peak of chlorogenic acid was detected at *m/z* 191.0 as [quinic acid−H]^−^, formed by the loss of the caffeoyl group in Q1 [20]. The Q3 ion peak for *p*-coumaric acid was detected at *m/z* 147.0 as [M + H − H_2_O]^+^, with the loss of a mass of a water molecule from the Q1 peak [21]. The Q3 peaks of 6-*C*-glycosides, orientin, and vitexin, and 8-*C*-glycosides, isoorientin, and isovitexin were detected at *m/z* 327.1, 311.1, 327.1, and 311.1, respectively. All the *C*-glycosides were detected as ^0,2^X^−^ ([M–H–C_4_H_8_O_4_]^−^) (Figure S2) forms in which water molecule was removed from the Q1 peak [22,23].

#### 2.2.2. Validation of the LC–MS/MS MRM Analytical Method

The developed LC–MS/MS MRM analytical method was validated with respect to the linearity, LOD, LOQ, accuracy, and precision. The validation results are summarized in Table 6, Table 7 and Table 8. The *r*^2^, LOD, and LOQ values of all analytical markers were >0.99, 0.80–16.20 ng/mL, and 2.40–48.60 ng/mL, respectively (Table 6). The recovery of the markers was calculated from Equation (3) to be 96.56–118.81% (Table 7), and an RSD of <4.0% for precision was established by using Equation (4) (Table 8). These results validate the analytical method developed for quality control of *P. pubescens* leaves using LC–MS/MS.

#### 2.2.3. Quantification of the Six Marker Components in *P. pubescens* Leaves by LC–MS/MS MRM Mode

The LC–MS/MS analysis method developed and validated for quality assessment of *P. pubescens* leaves using the six marker analytes was successfully applied to the analysis of 80% ethanol extract. Two phenylpropanoids (chlorogenic acid and *p*-coumaric acid) and four *C*-glycosides (isoorientin, orientin, vitexin, and isovitexin) were eluted at 10.17, 14.10, 18.27, 18.60, 20.56, and 21.12 min, respectively (Figure 2 and Figure S3). The amounts of marker substances in *P. pubescens* leaves are shown in Table 9, which were detected in concentrations of 0.12–19.20 mg/g.

## 3. Discussion

In the present study, two analytical methods, HPLC–DAD and LC–MS/MS, for simultaneous quantitation of major components in *P. pubescens* leaves were developed and validated. Various constituents such as flavonoids (e.g., isoorientin and isovitexin), coumarins (e.g., scopoletin and othole), and phenylpropanoids (caffeic acid and ferulic acid) have been isolated and reported as the main components of leaves of Bamboo species [1,2,3,4,5,6,7,8,9].

Among the various phytochemicals, we selected 10 components (chlorogenic acid, caffeic acid, isoorientin, orientin, isovitexin, vitexin, *p*-coumaric acid, ferulic acid, scopoletin, and tricin) HPLC–DAD analysis and attempted to analyze them using the water–acetonitrile (containing both 0.1% (*v/v*) formic acid) mobile-phase system. As a result, only the five components (chlorogenic acid, isoorientin, orientin, isovitexin, and *p*-coumaric acid) were detected (Figure S3), and these components were selected as markers for the development of a simultaneous analysis method for quality control of *P. pubescens* leaves using HPLC–DAD. Optimal analysis conditions were developed in analysis systems using the selected markers, and the developed method was verified with respect to the linearity, LOD, LOQ, accuracy, and precision. By using the developed and validated HPLC–DAD analytic method, all markers were eluted within 20.0 min (Figure 1). It was found that isoorientin was the most abundant in the established assay. In the analysis of *P. pubescens* leaves using HPLC reported by Wang et al. [9] and Jin et al. [24], flavone C-glycoside, isoorientin, was detected the most. These results show analysis results equivalent to those of our study.

Simultaneous quantitative analysis for quality control of *P. pubescens* leaves was performed using LC–MS/MS along with HPLC analysis. In order to select a marker analyte, LC–MS/MS MRM analysis was attempted on eight components (chlorogenic acid, caffeic acid, isoorientin, orientin, isovitexin, vitexin, *p*-coumaric acid, and ferulic acid) among various phytochemicals reported in *P. pubescens* leaves [1,2,3,4,5,6,7,8,9]. Six components (chlorogenic acid, *p*-coumaric acid, isoorientin, orientin, vitexin, and isovitexin) of them were detected in the *P. pubescens* leave sample (Figure S4), and these were selected as marker analytes for simultaneous analysis for quality control of *P. pubescens* leaves. An LC–MS/MS MRM method for the simultaneous quantification of *P. pubescens* leaves was developed using the selected markers, and this method was verified through linearity, LOD, LOQ, accuracy, and precision. Under the established LC–MS/MS MRM method, all markers were eluted within 22.0 min (Figure 2). Few studies have been reported on quantitative methods using LC–MS/MS. In the study reported by Wang et al., the LC–MS profile analysis was reported for four flavone *C*-glycoside components (isoorientin, orientin, vitexin, and isovitexin) [9], but no studies were conducted on quantitation and method validation. As a result of simultaneous analysis of *P. pubescens* leaves in the assay established in this study, isovitexin and isoorientin were found to have high concentrations of 19.20 mg/g and 9.33 mg/g, respectively.

Based on the above data, our study may be usefully applied to quality control of *P. pubescens* leaves in further studies.

## 4. Materials and Methods

### 4.1. Chemicals and Reagents

Authentic reference standard compounds (Figure S5) were purchased from natural product suppliers: chlorogenic acid (CAS No. 327-97-9, Catalog No. 109240010, C_16_H_18_O_9_, purity 99.6%) from Acros Organics (Pittsburgh, PA, USA); isoorientin (CAS No. 4261-42-1, Catalog No. DR11194, C_21_H_20_O_11_, purity 98.5%) from Shanghai Sunny Biotech (Shanghai, China); orientin (CAS No. 28608-75-5, Catalog No. BP1024, C_21_H_20_O_11_, purity 99.1%), isovitexin (CAS No. 38953-85-4, Catalog No. BP0804, C_21_H_20_O_10_, purity 99.3%); vitexin (CAS No. 3681-93-4, Catalog No. BP1447, C_21_H_20_O_10_, purity 99.7%) from Biopurify Phytochemicals (Chengdu, China); *p*-coumaric acid (CAS No. 501-98-4, Catalog No. 082-06521, C_9_H_8_O_3_, purity 99.2%) from Fujifilm Wako Pure Chemical Co. (Osaka, Japan). Solvents (methanol, acetonitrile, and water) used were HPLC-grade or LC–MS-grade and purchased from JT Baker (Phillipsburg, NJ, USA) or ThermoFisher Scientific (San Jose, CA, USA). Formic acid (CAS No. 64-18-6, Catalog No. 067-04531, purity 99.5%), dimethyl sulfoxide (DMSO, CAS No. 67-68-5, Catalog No. 472301, purity 99.9%), and ammonium formate (CAS No. 540-69-2, Catalog No. 70221, purity 99.0%) were LC–MS grade or ACS reagent grade and purchased from Fujifilm Wako Pure Chemical Co. (Osaka, Japan) or Merck KGaA (Darmstadt, Germany).

### 4.2. Plant Materials and Preparation of 80% Ethanol Extract of P. pubescens Leaves

Dried Chinese *P. pubescens* leaves (2020PPL) that are more than 3 years old were collected, dried naturally. The extract (production number: KOC-ZY-20191008) was processed by Zhenjiang KOC Biotech Co., Ltd. (Zhenjiang, China), a company specializing in herbal extracts. For the extraction process, the dried sample was extracted using 80% ethanol at 80 °C for 3 h and then filtered using a 100 mesh (150-μm) sieve. The extract was concentrated at 60 °C for 5 h under the pressure of 0.08–0.10 MPa and then dried with a microwave dryer, to obtain a powder sample in a yield of 10.0%.

### 4.3. HPLC–DAD Analytical Conditions

A Shimadzu Prominence LC-20A (Kyoto, Japan) linked to an SPD-M20A DAD was used as the HPLC system for simultaneous quantification of marker analytes in *P. pubescens* leaves. The system was controlled by LabSolution software (version 5.53, SP3, Kyoto, Japan). Analytical conditions such as analytical column, mobile phase, and gradient elution conditions for simultaneous quantification of marker components were determined according to a previous protocol [15]. The markers were quantified by measuring absorbance at 310 nm for *p*-coumaric acid, 325 nm for chlorogenic acid, 335 nm for isovitexin, and 350 nm for isoorientin and orientin using a DAD that simultaneously scanned from 190 to 400 nm. Details of the operating conditions are summarized in Table S1.

### 4.4. LC–MS/MS Analytical Conditions

The LC–MS/MS system consisted of an ACQUITY UPLC system (Waters, Milford, MA, USA) fitted with two pumps, a column oven, an auto-sampler, and a Xevo TQ-XS MS system coupled to an electrospray ionization source. The system was controlled by Waters MassLynx v4.2 software (Waters, Milford, MA, USA). The operating conditions used for UPLC and MS for quantitative analysis of *P. pubescens* leaves are summarized in Table S5, and conditions for the LC–MS/MS MRM analysis are shown in Table 5.

### 4.5. Preparation of Standard Solutions of Marker Analytes and Sample Solution

Marker analytes were accurately weighed and dissolved in methanol or methanol-DMSO (1:1) to a concentration of about 1.0 mg/mL and used as a standard solution. Each prepared standard stock solution was degassed in a sonicator and filtered through a 0.2 μm syringe filter (Pall Life Sciences, Ann Arbor, MI, USA). All stock solutions were stored in a refrigerator until the HPLC or LC–MS/MS analysis.

A sample solution for simultaneous analysis for quality control of *P. pubescens* leaves was prepared by dissolving 80% ethanol extract of *P. pubescens* leaves in 70% methanol at a concentration of 10 mg/mL. The solution was prepared by ultrasonic extraction for 60 min and then filtered through a 0.2 μm syringe filter (Pall Life Sciences, Ann Arbor, MI, USA). For the LC–MS/MS analysis, the prepared sample solution was diluted 10-fold prior to use.

### 4.6. Method Validation of Developed HPLC–DAD Assay

The developed HPLC–DAD analytical method was validated by testing linearity, range, LOD, LOQ, recovery, and precision. The linearity was established by determining the *r*^2^ value from the regression equation of the calibration curve prepared from a range of concentrations of each marker analyte: 0.31–20.00 μg/mL for chlorogenic acid, orientin, isovitexin, and *p*-coumaric acid and 0.78–50.00 μg/mL for isoorientin. LOD and LOQ were calculated from Equations (1) and (2) as follows:

(1) $$\mathrm{LOD}\;\left(\mu g/\mathrm{mL}\right)\;=\;3.3\times \frac{\sigma }{S}$$

(2) $$\mathrm{LOQ}\;\left(\mu g/\mathrm{mL}\right)\;=\;10\times \frac{\sigma }{S}$$

where *σ* and *S* are the standard deviation (SD) of the *y*-intercept and the slope of the calibration curve, respectively.

Recovery assays were used to establish accuracy. Thus, a known amount (low, medium, and high) of five markers was spiked into a sample and the recovered amount was calculated from Equation (3) as follows:

(3) $$\mathrm{Recovery}\;\left(\%\right)=\frac{\mathrm{Measured}\;\mathrm{amount}}{\mathrm{Spiked}\;\mathrm{amount}}\times 100$$

Precision was assessed with respect to repeatability, intra-day precision (within one day), and inter-day precision (successive three days), and reported as the RSD (%). Repeatability was evaluated by RSD (%) of retention time and peak area of each marker after six repeated measurements using a mixed standard solution. Intra-day and inter-day precision were also assessed by RSD (%) values. The RSD was calculated by Equation (4) as follows:

(4) $$\mathrm{RSD}\;\left(\%\right)=\frac{\mathrm{SD}}{\mathrm{Mean}}\times 100$$

### 4.7. Method Validation of the Developed LC–MS/MS MRM Assay

The LC–MS/MS MRM method was validated with respect to linearity, range, LOD, LOQ, accuracy, and precision, as described for the HPLC method (Section 4.6). The linearity was determined by the *r*^2^ of the calibration curves of each analyte prepared at different concentrations: 75.00–1200.00 ng/mL for chlorogenic acid and *p*-coumaric acid, 750.00–12,000.00 ng/mL for isoorientin, 200.00–3200.00 ng/mL for orientin and isovitexin, and 40.00–640.00 ng/mL for vitexin. LOD, LOQ, recovery, and precision were calculated and evaluated based on Equations (1)–(4), respectively.

### 4.8. Statistical Analysis

Data were presented as mean, SD, and RSD (%) by using Microsoft Excel 2019 software (Microsoft Co., Redmond, WA, USA).

## 5. Conclusions

We developed a method for the simultaneous analysis of major marker components in *P. pubescens* leaves based on widely used and convenient HPLC–DAD instrumentation and by using the fast, accurate, and sensitive the LC–MS/MS MRM method. Both methods were validated with respect to linearity, LOD, LOQ, recovery, and precision and met all required standards. Furthermore, the developed methods were successfully applied to the analysis of samples of *P. pubescens* leaves. Therefore, the analytical method described herein can be applied for quality control of *P. pubescens* leaves. In particular, the LC–MS/MS method will be useful for analyzing complex TCM, TKM, and KM containing *P. pubescens* leaves and for the study of pharmacokinetics and bioavailability using human plasma.

## Figures and Tables

**Table 1:** Parameters for simultaneous HPLC analysis (*n* = 3) of the five marker analytes in *P. pubescens* leaves extract.

Analyte	Quantification Wavelength (nm)	Linear Range (μg/mL)	Regression Equation ^a^ y=ax+b	*r* ^2^	LOD ^b^ (μg/mL)	LOQ ^c^ (μg/mL)
Chlorogenic acid	325	0.31–20.00	*y* = 34,768.31*x* + 404.96	1.0000	0.03	0.09
Isoorientin	350	0.78–50.00	*y* = 38,934.35*x* + 46.76	1.0000	0.10	0.29
Orientin	350	0.31–20.00	*y* = 35,868.34*x* + 13.07	1.0000	0.04	0.13
Isovitexin	335	0.31–20.00	*y* = 37,242.75*x* + 481.66	1.0000	0.04	0.13
*p*-Coumaric acid	310	0.31–20.00	*y* = 96,763.89*x* + 1244.28	1.0000	0.03	0.09

^a^ *y* and *x* represent the peak area and concentration of each marker analyte, respectively. ^b^ LOD means the limit of detection. ^c^ LOQ means the limit of quantification.

**Table 2:** Recovery (%) of the five marker analytes in the developed HPLC assay (*n* = 5).

Analyte	Spiked Conc. (μg/mL)	Measured Conc. (μg/mL)	Recovery (%)	SD	RSD (%)
Chlorogenic acid	1.00	0.99	99.23	2.06	2.08
	2.00	1.97	98.69	1.18	1.19
	4.00	3.93	98.21	0.51	0.52
Isoorientin	2.00	1.90	95.22	1.39	1.46
	5.00	4.90	97.94	0.72	0.74
	10.00	9.83	98.27	0.73	0.75
Orientin	1.00	0.97	96.72	1.56	1.61
	2.00	1.93	96.66	1.52	1.57
	4.00	3.89	97.30	0.28	0.29
Isovitexin	1.00	0.98	98.05	2.18	2.22
	2.00	1.95	97.69	1.47	1.50
	4.00	3.88	96.99	1.29	1.33
*p*-Coumaric acid	1.00	1.01	101.17	0.93	0.92
	2.00	2.03	101.29	0.96	0.95
	4.00	3.94	98.40	1.58	1.61

**Table 3:** Precision of the developed HPLC analysis method using 5 markers.

Analyte	Conc. (μg/mL)	Intra-Day (*n* = 5)			Inter-Day (*n* = 5)		
		Measured Conc. (μg/mL)	Precision (RSD, %)	Accuracy (%)	Measured Conc. (μg/mL)	Precision (RSD, %)	Accuracy (%)
Cholrogenic acid	5.00	5.02	0.52	100.41	5.09	1.38	101.75
	10.00	10.03	0.92	100.29	10.14	1.24	101.43
	20.00	20.03	0.37	100.17	20.33	1.24	101.64
Isoorientin	12.50	12.56	0.53	100.47	12.75	1.48	102.00
	25.00	25.06	0.83	100.24	25.36	1.26	101.44
	50.00	50.08	0.37	100.15	50.88	1.36	101.76
Orientin	5.00	5.01	0.62	100.28	5.09	1.50	101.81
	10.00	10.00	0.68	100.01	10.13	1.21	101.27
	20.00	20.02	0.25	100.12	20.34	1.35	101.72
Isovitexin	5.00	5.04	0.31	100.80	5.11	1.39	102.22
	10.00	10.03	0.75	100.34	10.15	1.24	101.50
	20.00	20.04	0.31	100.21	20.36	1.35	101.82
*p*-Coumaric acid	5.00	5.02	0.36	100.48	5.10	1.40	102.00
	10.00	10.03	0.78	100.33	10.15	1.25	101.51
	20.00	20.02	0.28	100.11	20.33	1.29	101.67

**Table 4:** Quantitation of the five marker compounds in samples of *P. pubescens* leaves by HPLC–DAD (*n* = 3).

Compound	Batch 1			Batch 2			Batch 3		
	Mean (mg/g)	SD × 10^−1^	RSD (%)	Mean (mg/g)	SD × 10^−1^	RSD (%)	Mean (mg/g)	SD × 10^−1^	RSD (%)
Chlorogenic acid	1.71	0.08	0.44	1.85	0.16	0.84	1.78	0.17	0.95
Isoorientin	10.94	0.18	0.16	11.63	0.23	0.20	11.29	0.30	0.27
Orientin	3.06	0.03	0.09	3.25	0.09	0.29	3.15	0.02	0.07
Isovitexin	4.36	0.15	0.34	4.65	0.06	0.12	4.51	0.09	0.20
*p*-Coumaric acid	2.13	0.04	0.17	2.27	0.07	0.30	2.20	0.10	0.44

**Table 5:** LC–MS/MS MRM transitions for quantitative analysis of markers in *P. pubescens* leaves.

Analyte	Ion Mode	Molecular Weight	MRM Transition	Cone Voltage (V)	Collision Energy (eV)	Retention Time (min)
Chlorogenic acid	−	354.1	353.2 → 191.0	20	20	10.17
*p*-Coumaric acid	+	164.0	165.0 → 147.0	20	10	14.10
Isoorientin	−	448.1	447.2 → 327.1	45	25	18.27
Orientin	−	448.1	447.2 → 327.1	45	25	18.60
Vitexin	−	432.1	431.2 → 311.1	45	15	20.56
Isovitexin	−	432.1	431.2 → 311.1	45	15	21.12

**Table 6:** Linear range, regression equation, *r*^2^, LOD, and LOQ for simultaneous analysis of marker analytes in *P. pubescens* leaves using LC–MS/MS MRM mode.

Analyte	Linear Range (ng/mL)	Regression Equation ^a^ y=ax+b	*r* ^2^	LOD (ng/mL)	LOQ (ng/mL)
Chlorogenic acid	75.00–1200.00	*y* = 125.51*x* − 880.85	0.9976	3.00	8.90
*p*-Coumaric acid	75.00–1200.00	*y* = 81.33*x* + 258.47	0.9972	12.00	36.10
Isoorientin	750.00–1200.00	*y* = 137.42*x* + 109,104.00	0.9954	1.00	3.00
Orientin	200.00–3200.00	*y* = 62.79*x* + 1809.43	0.9980	14.90	44.80
Vitexin	40.00–640.00	*y* = 277.01*x* + 773.67	0.9998	0.80	2.40
Isovitexin	200.00–3200.00	*y* = 12.27*x* − 7.50	0.9998	16.20	48.60

^a^*y*: peak area of compounds; *x*: concentration of compounds.

**Table 7:** Extract recovery tests for 6 marker components in *P. pubescens* leaves.

Analyte	Spiked Amount (ng/mL)	Found Amount (ng/mL)	Recovery (%)	SD	RSD (%)
Chlorogenic acid	300	299.56	99.85	2.62	2.62
	600	623.52	103.92	1.21	1.17
	1200	1259.64	104.97	1.58	1.50
*p*-Coumaric acid	300	296.46	98.82	4.48	4.54
	600	627.02	104.50	4.42	4.23
	1200	1255.80	104.65	2.41	2.30
Isoorientin	3000	3151.78	105.06	2.33	2.22
	6000	7128.88	118.81	1.11	0.94
	12,000	13,405.70	111.71	2.00	1.79
Orientin	800	798.62	99.83	1.32	1.32
	1600	1689.00	105.56	1.25	1.18
	3200	3395.94	106.12	2.12	2.00
Vitexin	160	154.50	96.56	2.21	2.29
	320	332.22	103.82	2.13	2.05
	640	681.10	106.42	2.20	2.07
Isovitexin	800	778.50	97.31	2.58	2.65
	1600	1608.16	100.51	2.45	2.44
	3200	3331.50	104.11	1.25	1.20

**Table 8:** Precision data of LC–MS/MS MRM assay for 6 marker components in *P. pubescens* leaves.

Analyte	Conc. (μg/mL)	Intraday (*n* = 5)				Interday (*n* = 5)		Repeatability (*n* = 6)	
		Observed Conc. (μg/mL)	Precision (RSD, %)	Accuracy (%)	Observed Conc. (μg/mL)	Precision (RSD, %)	Accuracy (%)	RSD (%) of Retention Time	RSD (%) of Peak Area
Chlorogenic acid	300	281.30	1.96	93.77	297.70	2.00	99.23	0.07	0.61
	600	615.06	0.38	102.51	624.70	0.61	104.12		
	1200	1289.38	0.48	107.45	1264.40	0.90	105.37		
*p*-Coumaric acid	300	311.40	1.95	103.71	302.00	3.61	100.66	0.08	3.76
	600	630.24	2.21	105.04	620.10	2.60	103.35		
	1200	1259.64	3.03	104.97	1249.6	3.00	104.13		
Isoorientin	3000	2989.90	3.03	99.66	3107.00	2.54	103.57	0.02	0.73
	6000	6894.80	0.65	114.91	7017.80	0.71	116.96		
	12,000	13,647.06	1.48	113.73	13,531.4	1.58	112.76		
Orientin	800	760.34	2.21	95.04	776.50	1.38	97.06	0.08	1.94
	1600	1660.46	3.78	103.78	1674.90	2.10	104.68		
	3200	3514.78	1.33	109.84	3421.20	1.27	106.91		
Vitexin	160	153.32	3.85	95.83	154.80	2.29	96.77	0.03	0.93
	320	335.36	2.88	104.80	334.90	2.50	104.65		
	640	696.22	1.21	108.78	683.30	1.43	106.76		
Isovitexin	800	724.14	1.52	90.52	762.90	1.58	95.37	0.03	0.82
	1600	1657.38	3.15	106.59	1654.10	2.08	103.38		
	3200	3476.34	1.34	108.64	3362.30	1.01	105.07		

**Table 9:** Amounts of the six marker analytes in *P. pubescens* leaves determined by the LC–MS/MS MRM method (*n* = 3).

Analyte	Amount		
	Mean (mg/g)	SD (×10^−1^)	RSD (%)
Chlorogenic acid	1.74	0.03	0.17
*p*-Coumaric acid	1.76	0.26	1.49
Isoorientin	9.33	0.35	0.37
Orientin	3.95	0.24	0.62
Vitexin	0.12	0.04	3.50
Isovitexin	19.20	1.00	0.52

## Data Availability

The data presented in this study are available in the article (tables and figures).

## Supplementary Materials

The following are available online at https://www.mdpi.com/article/10.3390/plants11010050/s1, Figure S1: Extracted ion chromatograms of each standard marker (A) and marker compound in 80% ethanol extract of the microwave-dried *P. pubescens* leaves sample (B) measured by LC–MS/MS MRM mode, Figure S2: Fragmentation of the *C*-glycosides, Figure S3: Chromatogram standard solution (A), *P. pubescens* leaves sample (B) for selecting marker components of *P. pubescens* leaves by HPLC–DAD analysis system. Chlorogenic acid (**1**), caffeic acid (**2**), isoorientin (**3**), orientin (**4**), isovitexin (**5**), vitexin (**6**), *p*-coumaric acid (**7**), ferulic acid (**8**), scopoletin (**9**), and tricin (**10**), Figure S4: Total ion chromatogram standard solution (A), *P. pubescens* leaves sample (B) for selecting marker components of *P. pubescens* leaves by LC–MS/MS MRM analysis system in positive and negative ion modes. Chlorogenic acid (**1**), caffeic acid (**2**), *p*-coumaric acid (**3**), ferulic acid (**4**), isoorientin (**5**), orientin (**6**), vitexin (**7**), and isovitexin (**8**), Figure S5: Chemical structures of the six marker components in *P. pubescens* leaves, Table S1: Chromatographic parameters for simultaneous analysis of five marker components in *P. pubescens* leaves by HPLC, Table S2: System suitability for HPLC analysis of the five marker components, Table S3: Repeatability of retention time of the five marker analytes using HPLC (*n* = 6), Table S4: Repeatability of peak area of the five marker analytes using HPLC (*n* = 6), Table S5: LC–MS/MS MRM analysis conditions for quantification of markers in *P. pubescens* leaves.

## References

[1] Wang S., Tang F., Yue Y., Tao X., Wei Q., Yu J. (2013). Simultaneous determination of 12 coumarins in bamboo leaves by HPLC. J. AOAC Int..

[2] Kim A., Im M., Gu M.J., Ma J.Y. (2016). Ethanol extract of *Lophatheri Herba* exhibits anti-cancer activity in human cancer cells by suppression of metastatic and angiogenic potential. Sci. Rep..

[3] Ma N.H., Guo J., Chen S.H., Yuan X.R., Zhang T., Ding Y. (2020). Antioxidant and compositional HPLC analysis of three common bamboo leaves. Molecules.

[4] Zhang Y., Bao B., Lu B., Ren Y., Tie X., Zhang Y. (2005). Determination of flavone C-glucosides in antioxidant of bamboo leaves (AOB) fortified foods by reversed-phase high-performance liquid chromatography with ultraviolet diode array detection. J. Chromatogr. A.

[5] Wang L., Bai M., Qin Y., Liu B., Wang Y., Zhou Y. (2018). Application of ionic liquid-based ultrasonic-assisted extraction of flavonoids from bamboo leaves. Molecules.

[6] Ma Y., Zhu D., Wang C., Zhang Y., Shang Y., Liu F., Ye T., Chen X., Wei Z. (2018). Simultaneous and fast separation of three chlorogenic acids and two flavonoids from bamboo leaves extracts using zirconia. Food Chem. Toxicol..

[7] Wang C.Z., Zhang H.Y., Li W.J., Ye J.Z. (2015). Chemical constituents and structural characterization of polysaccharides from four typical bamboo species leaves. Molecules.

[8] Sun Y., Yang K., Cao Q., Sun J., Xia Y., Wang Y., Li W., Ma C., Liu S. (2017). Homogenate-assisted vacuum-powered bubble extraction of moso bamboo flavonoids for on-line scavenging free radical capacity analysis. Molecules.

[9] Wang J., Yue Y., Jiang H., Tang F. (2012). Rapid screening for flavone *C*-glycosides in the leaves of different species of bamboo and simultaneous quantitation of four marker compounds by HPLC-UV/DAD. Int. J. Anal. Chem..

[10] Kwon J.H., Hwang S.Y., Han J.S. (2017). Bamboo (*Phyllostachys bambusoides*) leaf extracts inhibit adipogenesis by regulating adipogenic transcription factors and enzymes in 3T3-L1 adipocytes. Food Sci. Biotechnol..

[11] Liao M., Ren X., Gao Q., Liu N., Tang F., Wang G., Cao H. (2021). Anti-fungal activity of moso bamboo (*Phyllostachys pubescens*) leaf extract and its development into a botanical fungicide to control pepper phytophthora blight. Sci. Rep..

[12] Kim D.S., Kim S.H., Cha J. (2016). Antiobesity effects of the combined plant extracts varying the combination ratio of *Phyllostachys pubescens* leaf extract and *Scutellaria baicalensis* root extract. Evid. Based Complement. Alternat. Med..

[13] Cho E., Kim S., Na I., Dim D.C., In M.J., Chae H.J. (2010). Antioxidant and anticoagulant activities of water and ethanol extracts of *Phyllostachys pubescence* leaf produced in Geoje. J. Appl. Biol. Chem..

[14] Shen Z.H., Hu X.Q., Hu M.J., Pan X.K., Lu H.G., Chen B., Wu B., Chen G. (2020). Activation of AKT signaling via small molecule natural compound prevents against osteoblast apoptosis and osteonecrosis of the femoral head. Am. J. Transl. Res..

[15] Song K.H., Seo C.S., Yang W.K., Gu H.O., Kim K.J., Kim S.H. (2021). Extracts of Phyllostachys pubescens leaves represses human steroid 5-alpha reductase type 2 promoter activity in BPH-1 cells and ameliorates testosterone-induced benign prostatic hyperplasia in rat model. Nutrients.

[16] Jin Y.C., Yin L., Ke Y. (2012). A novel high-performance liquid chromatography fingerprint approach to discriminate *Phyllostachys pubescens* from China. Pharmacogn. Mag..

[17] Liang F., Wang R., Xiang H., Tang X., Zhang T., Hu W., Mi B., Liu Z. (2018). Investigating pyrolysis characteristics of moso bamboo through TG-FTIR and Py-GC/MS. Bioresour. Technol..

[18] Pang B., Zhu Y., Lu L., Gu F., Chen H. (2016). The applications and features of liquid chromatography-mass spectrometry in the analysis of traditional Chinese medicine. Evid. Based Complement. Altern. Med..

[19] Wang X., Zhang A., Yan G., Han Y., Sun H. (2014). UHPLC-MS for the analytical characterization of traditional Chinese medicines. TrAC Trends Anal. Chem..

[20] Jaiswal R., Müller H., Müller A., Karar M.G.E., Kuhnert N. (2014). Identification and characterization of chlorogenic acids, chlorogenic acid glycosides and flavonoids from *Lonicera henryi* L. (Caprifoliaceae) leaves by LC-MS^n^. Phytochemistry.

[21] Wang J., Yue Y.D., Tang F., Sun J. (2012). Screening and analysis of the potential bioactive components in rabbit plasma after oral administration of hot-water extracts from leaves of *Bambusa textilis* McClure. Molecules.

[22] Yao H., Chen Y., Shi P., Hu J., Li S., Huang L., Lin J., Lin X. (2012). Screening and quantitative analysis of antioxidants in the fruits of *Livistona chinensis* R. Br using HPLC-DAD-ESI/MS coupled with pre-column DPPH assay. Food Chem..

[23] Li X., Xiong Z., Ying X., Cui L., Zhu W., Li F. (2006). A rapid ultra-performance liquid chromatography-electrospray ionization tandem mass spectrometric method for the qualitative and quantitative analysis of the constituents of the flower of *Trollius ledibouri* Reichb. Anal. Chim. Acta.

[24] Jin Y., Liu H., Yuan K. (2011). Simultaneous determination of seven effective constituents in the leaves of bamboo by reversed phase high performance liquid chromatography (RP-HPLC). J. Med. Plants Res..

