# Supplementary material for: Phytochemical Characterization for Quality Control of Phyllostachys pubescens Leaves Using High-Performance Liquid Chromatography Coupled with Diode Array Detector and Tandem Mass Detector
Source: Plants (Basel). 2021 Dec 24;11(1):50. doi: 10.3390/plants11010050 (PMC8747080; doi:10.3390/plants11010050)
Supplement: Supplementary file 1 [file plants-11-00050-s001.zip › plants-1514487-supplementary.pdf]

Table S1

Chromatographic parameters for simultaneous analysis of five marker components in *P. pubescens* leaves by HPLC

Chromatographic parameter			
Column	Gemini C_{18} analytical column (250 mm × 4.6 mm, 5 μm)		
Detector	DAD (310, 325, 335, and 350 nm)		
Flow rate	1.0 mL/min		
Injection volume	10.0 μL		
Column temperature	40.0 °C		
Mobile phase	A: 0.1% (v/v) aqueous formic acid<br>B: 0.1% (v/v) formic acid in acetonitrile		
Gradient elution	Time (min)	A (%)	B (%)
	0	95	5
	25	40	60
	30	60	60
	35	95	5
	45	95	5

DAD; diode array detector

Table S2

System suitability for HPLC analysis of the five marker components

Compound	k'	α	N	Rs	T_f
Chlorogenic acid	2.94	1.19	399752	14.09	1.08
Isoorientin	3.50	1.19	650618	3.46	1.10
Orientin	3.63	1.04	633528	3.46	1.08
Isovitexin	3.95	1.03	722500	2.62	1.06
p-Coumaric acid	4.06	1.03	529916	2.62	1.05

k'; capacity factor, α; relative retention, N; theoretical plate number, Rs; resolution, and T_f; tailing factor

Table S3

Repeatability of retention time of the five marker analytes using HPLC (n = 6)

No.	Retention time (min)				
	Chlorogenic acid	Isoorientin	Orientin	Isovitexin	*p*-Coumaric acid
1	12.23	13.91	14.33	15.29	15.65
2	12.24	13.92	14.34	15.31	15.66
3	12.24	13.92	14.34	15.31	15.66
4	12.24	13.92	14.34	15.30	15.66
5	12.24	13.92	14.34	15.31	15.66
6	12.25	13.93	14.34	15.31	15.66
Mean	12.24	13.92	14.34	15.30	15.66
SD	0.01	0.01	0.01	0.01	0.01
RSD (%)	0.06	0.04	0.04	0.04	0.04

Table S4

Repeatability of peak area of the five marker analytes using HPLC (n = 6)

No.	Peak area				
	Chlorogenic acid	Isoorientin	Orientin	Isovitexin	*p*-Coumaric acid
1	1,770,950	1,506,998	1,292,847	1,423,144	1,897,993
2	1,767,368	1,502,200	1,288,098	1,418,291	1,890,987
3	1,794,637	1,525,428	1,306,379	1,438,257	1,918,884
4	1,768,468	1,505,733	1,287,574	1,419,711	1,893,611
5	1,780,640	1,519,401	1,298,785	1,432,187	1,909,363
6	1,791,903	1,528,323	1,309,987	1,443,697	1,924,334
Mean	1,778,994.33	1,514,680.50	1,297,278.33	1,429,214.50	1,905,862.00
SD	12,039.28	11,124.31	9430.71	10,457.87	13,829.81
RSD (%)	0.68	0.73	0.73	0.73	0.73

Table S5

LC–MS/MS MRM analysis conditions for quantification of markers in *P. pubescens* leaves

**UPLC conditions**		**MS conditions**	
UPLC system	Acquity UPLC I-Class	MS system	Xevo TQ-XS
Column	Acquity UPLC BEH C_{18} column (2.1 mm × 100 mm, 1.7 µm)	MS software	MassLynx v4.2
Column temp.	45 °C	Ion source	ESI^+ or ESI^-
Sample temp.	5 °C	Acquisition mode	MRM
Injection volume	2.0 µL	Capillary voltage	3.0 kV
Flow rate	0.3 mL/min	Cone gas flow	50 L/h
Mobile phase A	0.1% (v/v) formic acid and 5 mM ammonium formate in distilled water	Desolvation gas flow	700 L/h
Mobile phase B	Acetonitrile	Desolvation temp.	500 °C
Gradient	(see below)	Source temp.	150 °C

Gradient:

Time (min)	A (%)	B (%)
Initial	100	0
23	85	15
23.1	5	95
24.9	5	95
25.0	100	0
28.0	100	0

ESI; electrospray ionization, MRM; multiple reaction monitoring

(A)

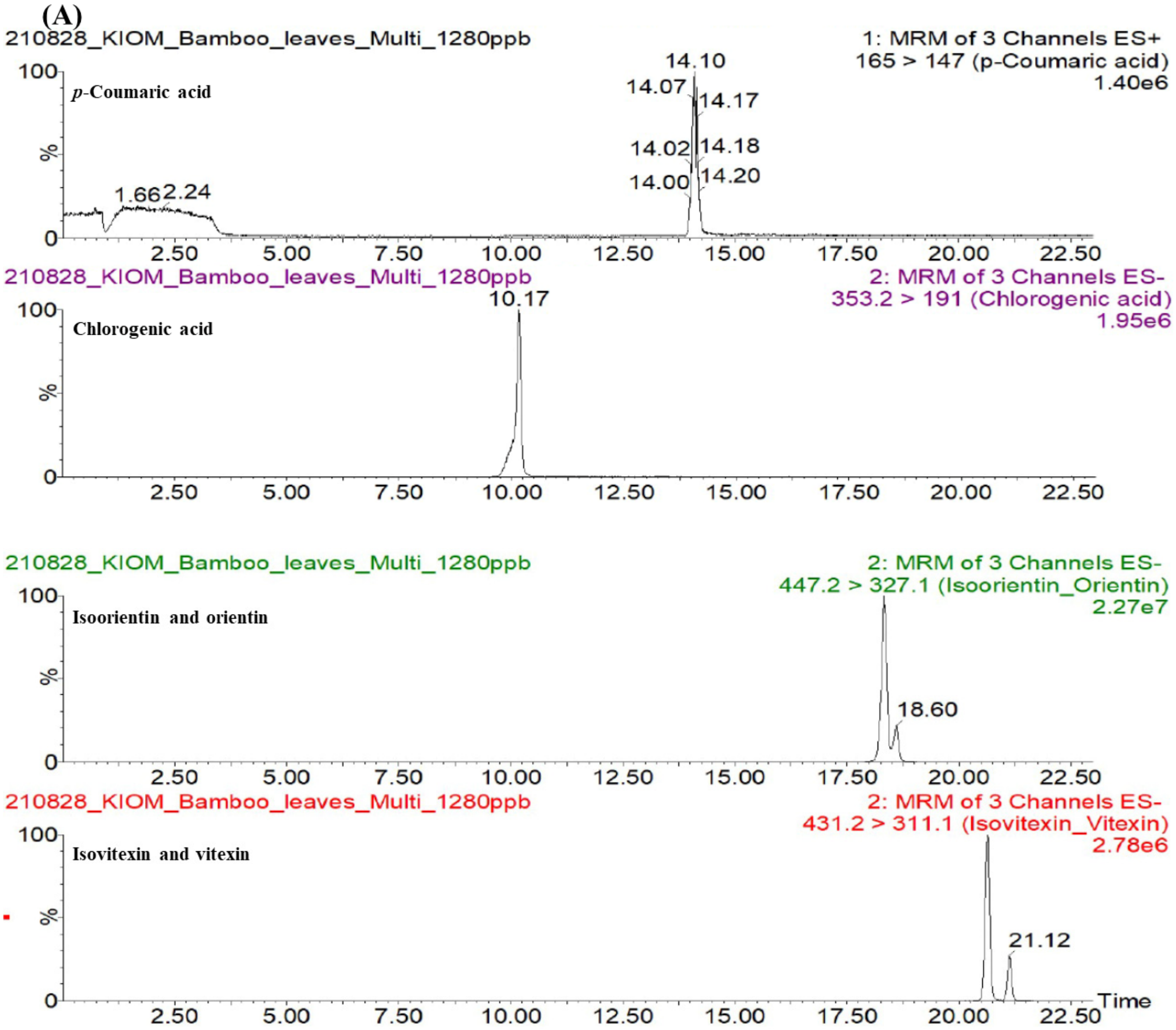
210828_KIOM_Bamboo_leaves_Multi_1280ppb
1: MRM of 3 Channels ES+
165 > 147 (p-Coumaric acid)
1.40e6
p-Coumaric acid
14.10
14.07
14.17
14.02
14.18
14.00
14.20
1.66
2.24
210828_KIOM_Bamboo_leaves_Multi_1280ppb
2: MRM of 3 Channels ES-
353.2 > 191 (Chlorogenic acid)
1.95e6
Chlorogenic acid
10.17
210828_KIOM_Bamboo_leaves_Multi_1280ppb
2: MRM of 3 Channels ES-
447.2 > 327.1 (Isoorientin_Orientin)
2.27e7
Isoorientin and orientin
18.60
210828_KIOM_Bamboo_leaves_Multi_1280ppb
2: MRM of 3 Channels ES-
431.2 > 311.1 (Isovitexin_Vitexin)
2.78e6
Isovitexin and vitexin
21.12
100
%
0
2.50
5.00
7.50
10.00
12.50
15.00
17.50
20.00
22.50
Time

(B)

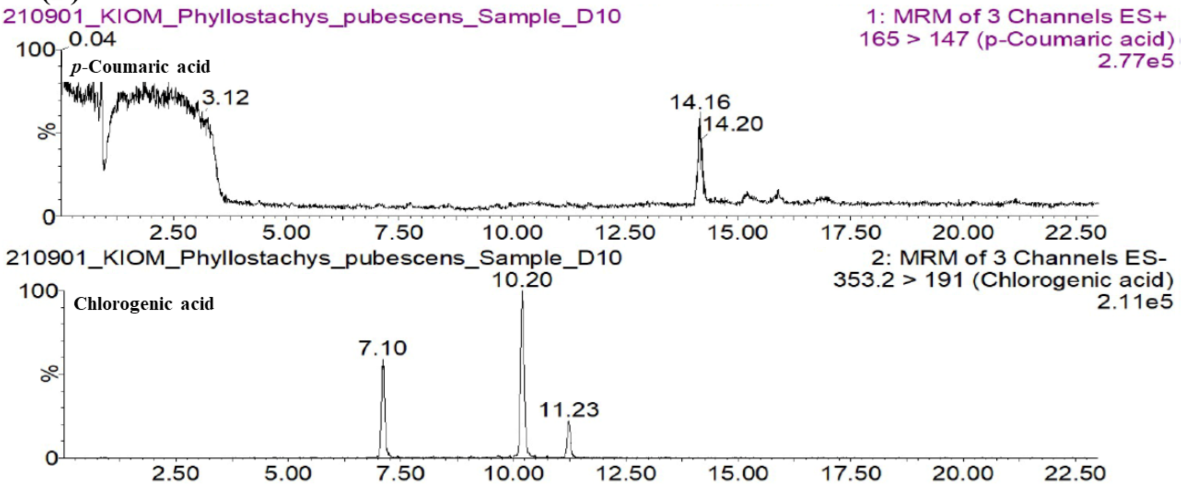
210901_KIOM_Phyllostachys_pubescens_Sample_D10
1: MRM of 3 Channels ES+
165 > 147 (p-Coumaric acid)
2.77e5
p-Coumaric acid
0.04
3.12
14.16
14.20
210901_KIOM_Phyllostachys_pubescens_Sample_D10
2: MRM of 3 Channels ES-
353.2 > 191 (Chlorogenic acid)
2.11e5
Chlorogenic acid
10.20
7.10
11.23
100
%
0
2.50
5.00
7.50
10.00
12.50
15.00
17.50
20.00
22.50

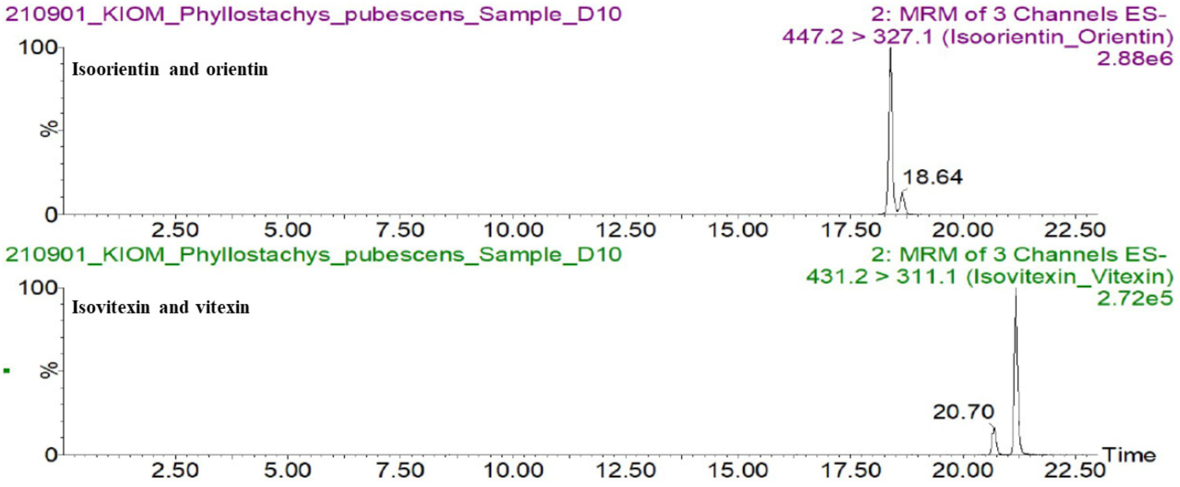

Figure S1. Extracted ion chromatograms of each standard marker (A) and marker compound in 80% ethanol extract of the microwave-dried *P. pubescens* leaves sample (B) measured by LC–MS/MS MRM mode.

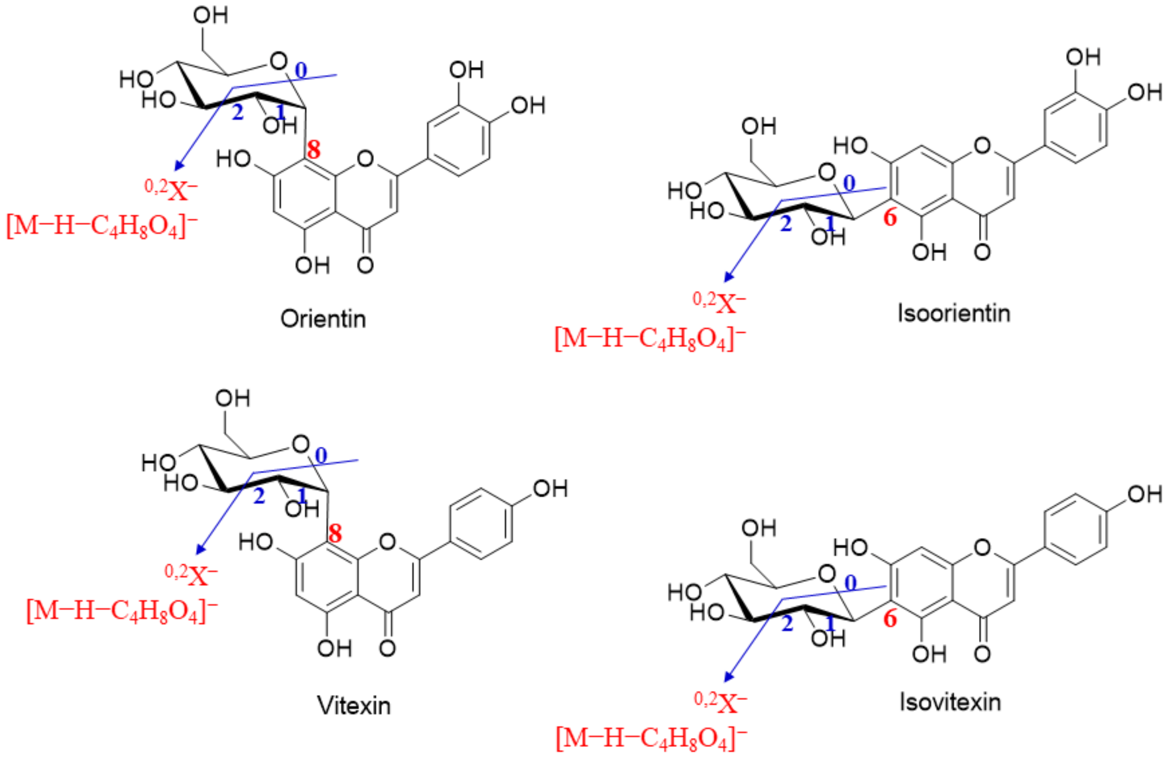

Figure S2. Fragmentation of the *C*-glycosides

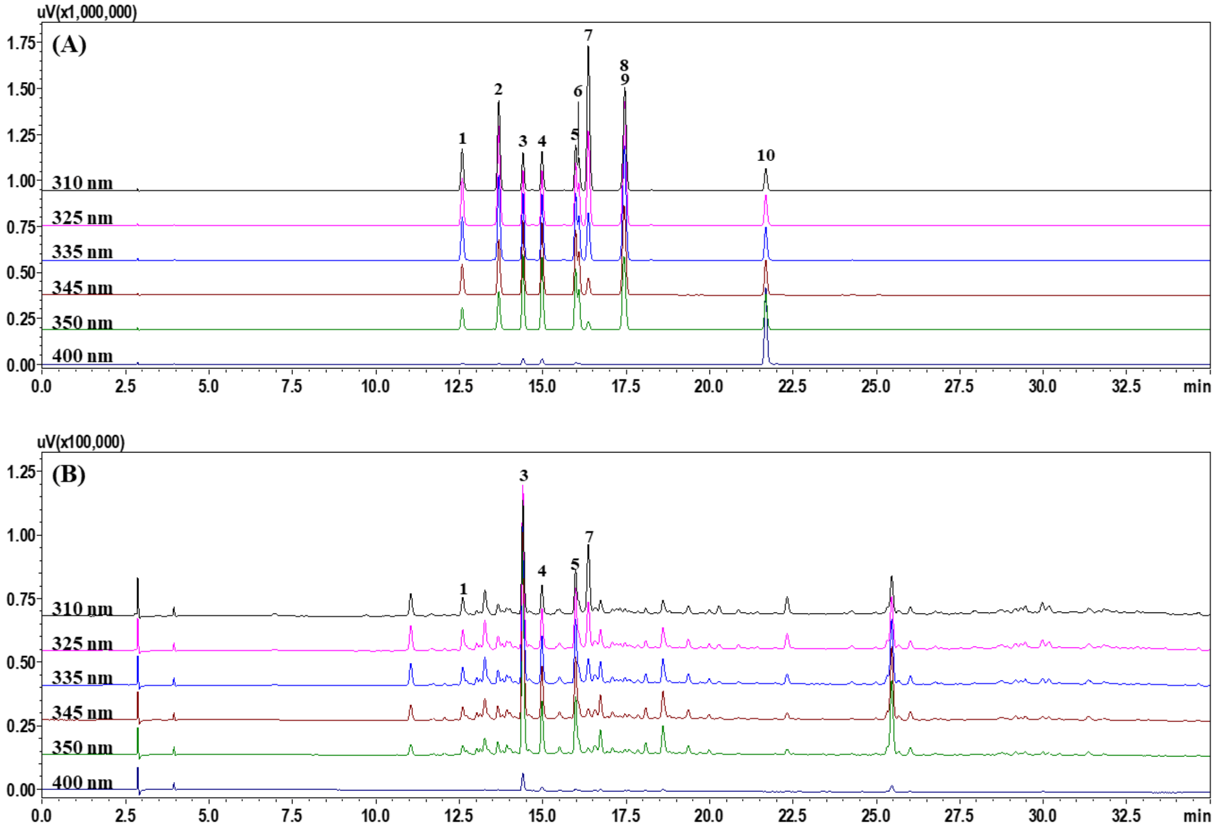

Figure S3. Chromatogram standard solution (A), *P. pubescens* leaves sample (B) for selecting marker components of *P. pubescens* leaves by HPLC–DAD analysis system. Chlorogenic acid (**1**), caffeic acid (**2**), isoorientin (**3**), orientin (**4**), isovitexin (**5**), vitexin (**6**), *p*-coumaric acid (**7**), ferulic acid (**8**), scopoletin (**9**), and tricin (**10**).

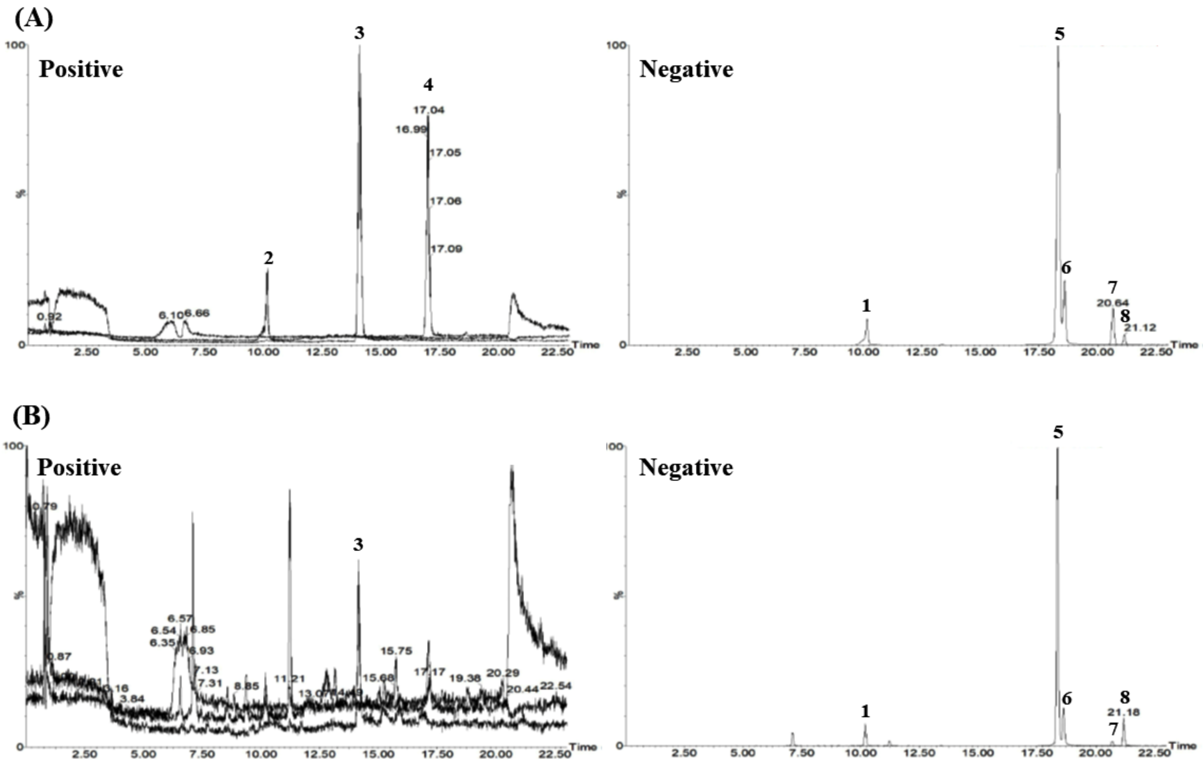

Figure S4. Total ion chromatogram standard solution (A), *P. pubescens* leaves sample (B) for selecting marker components of *P. pubescens* leaves by LC–MS/MS MRM analysis system in positive and negative ion modes. Chlorogenic acid (**1**), caffeic acid (**2**), *p*-coumaric acid (**3**), ferulic acid (**4**), iosorientin (**5**), orientin (**6**), vitexin (**7**), and isovitexin (**8**).

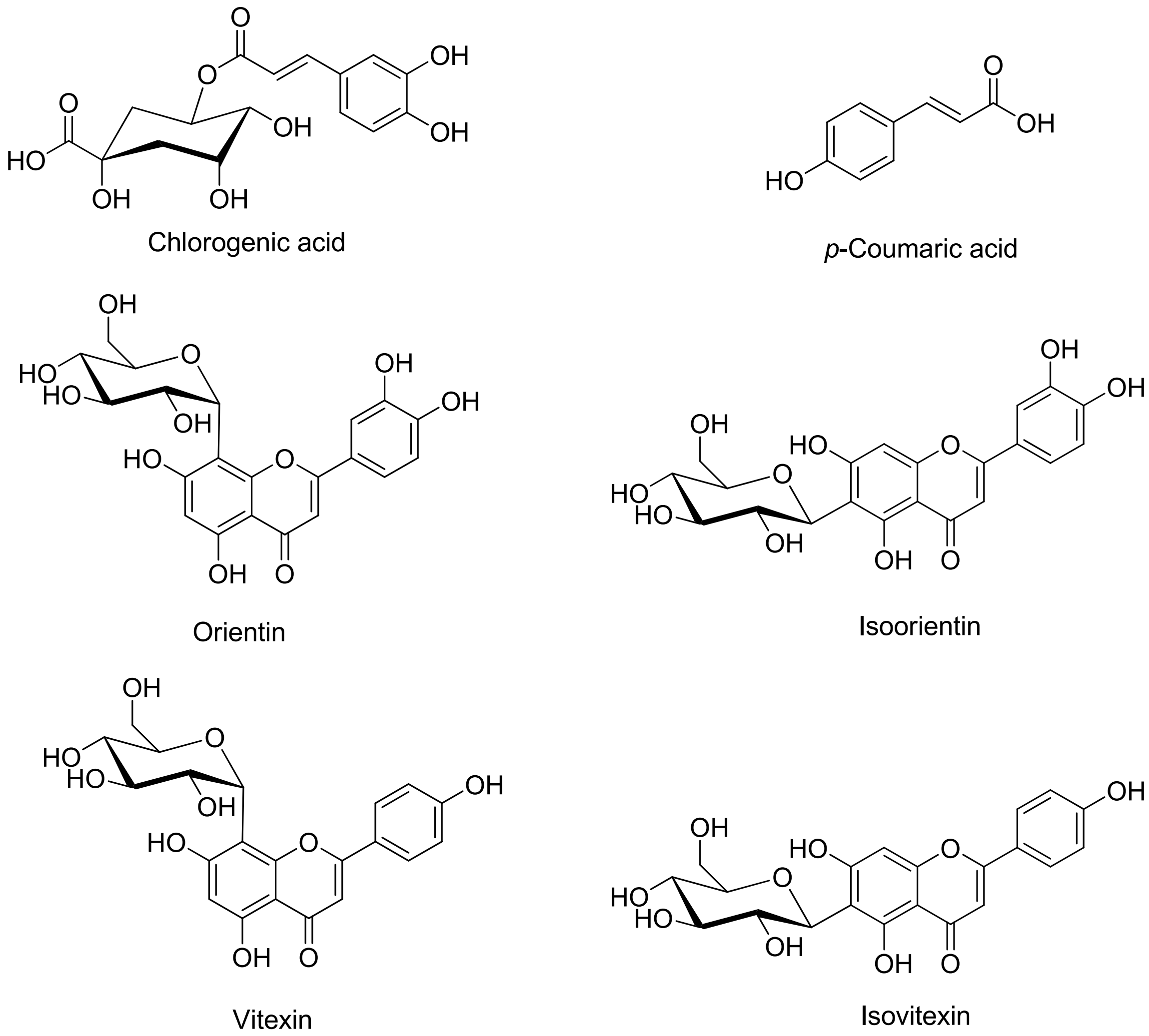

Figure S5. Chemical structures of the six marker components in *P. pubescens* leaves
